# Catalpol promotes hippocampal neurogenesis and synaptogenesis in rats after multiple cerebral infarctions by mitochondrial regulation: involvement of the Shh signaling pathway

**DOI:** 10.3389/fphar.2024.1461279

**Published:** 2024-12-19

**Authors:** Zishan Huang, Feng Li, Xiaoyu Zheng, Jiarui Zheng, Yilei Dong, Zhao Ding, Huanyu Gou, Mingjiang Yao, Jianxun Liu

**Affiliations:** ^1^ Institute of Basic Medical Sciences of Xiyuan Hospital, China Academy of Chinese Medical Sciences, Beijing Key Laboratory of Chinese Materia Pharmacology, National Clinical Research Center of Traditional Chinese Medicine for Cardiovascular Diseases, Beijing, China; ^2^ Institute of Chinese Medicine Sciences, Guangdong Pharmaceutical University, Guangzhou, China; ^3^ Heilongjiang Academy of Chinese Medicine, Harbin, Heilongjiang, China

**Keywords:** catalpol, Shh signaling pathway, mitochondria, cerebral infarction, neuro-restoration

## Abstract

**Introduction:**

Ischemic stroke greatly threatens human life and health. Neuro-restoration is considered to be the critical points in reestablishing neurological function and improving the quality of life of patients. Catalpol is the main active ingredient of the Chinese herbal medicine *Dihuang*, which has the beneficial efficacy in traditional remedy, is closely related to the mitochondrial morphology and function. In the present study, we investigated whether catalpol has a neurorestorative effect after multiple cerebral infarctions and its underlying mechanisms.

**Methods:**

In this study, male 8-week-old Sprague-Dawley (SD) rats were grouped according to neurological deficit scores to minimize differences between groups the second day: sham group, model group, Ginkgo biloba P.E (EGb) (Ginaton:18 mg/kg) group, model + CAT 30 mg/kg group (CAT 30), model + CAT 60 mg/kg group (CAT 60), and model + CAT 120 mg/kg group (CAT 120). From the first day to the fourteenth day after MCI, rats were given the corresponding doses of drugs by gastric administration every day(1 mL/100g), and from day 7 to day 14, all rats were injected with Brdu solution (50 mg/kg) i.p. Neuro-Function was assessed by the neurologic deficit scores. Then we observed measurement of brain atrophy and fluorescent Nissl staining. The expression of BrdU+/DCX+ cells and the BDNF concentrations were tested to observe the neuro-restoration effect. Transmission electron microscope (TEM) and Western blot (WB) were used to observed synaptogenesis. we observed the restoration of mitochondrial function by detecting the intracortical calcium and T-AOC content. Finally, we examined the protein and mRNA expression of shh signaling pathway through q-PCR and WB.

**Results:**

Catalpol alleviated neurological deficits, reduced the degree of brain atrophy, as well as minimize pathological damage in the hippocampus and cortex. In addition, catalpol also promoted hippocampal neurogenesis and synaptogenesis by improving the mitochondrial structure and promoting mitochondrial function, as evidenced by the up-regulation of positive expression of both Recombinant Doublecortin (DCX) and 5-Bromodeoxyuridinc (BrdU), the enhancement of the Total antioxidant capacity (T-AOC), and the increase in the expression of synapse-associated proteins, Synaptophysin (SYP) and post-synaptic density-95 (PSD-95). Finally, we observed that catalpol up-regulated the expression of Sonic hedgehog (Shh) and Glioma-associated homologue-1 (GLI-1), factors related to the Shh signaling pathway.

**Discussion:**

In conclusion, catalpol may regulate mitochondria through activation of the Shh signaling pathway and exert its role in promoting hippocampal neurogenesis and synaptogenesis.

## Highlight

• Catalpol promotes neuro-restoration processes that may involve mitochondria, including restoration of its structure and amelioration of dysfunction.
• The Shh signaling pathway is primarily associated with embryonic development, but may also involve mitochondrial in its effects.
• Catalpol may promote endogenous neural stem cell proliferation and differentiation, as well as synaptogenesis, through activation of the Shh signaling pathway.

## Introduction

Ischemic stroke is the temporary or permanent reduction of blood supply to brain tissue, resulting in local ischemia and hypoxia, which causes necrosis in different ranges of the brain and outwardly manifests itself in corresponding neurological function loss. Ischemic stroke accounts for about 80% of all strokes, and is characterized by high morbidity, disability, and mortality, which brings a heavy burden to the family and whole society (Saini et al., 2021). Revascularization is regarded as the routine treatment in the acute phase of ischemic stroke, by recanalization or using recombinant tissue Plasminogen Activator (rt-PA). However, such principles are quite difficult to implement in clinical practice because of the narrow time window (Sommer and Schäbitz, 2021), as well as side effects including reperfusion injury or hemorrhagic transformation. Furthermore, the antiplatelet and anticoagulant therapy, the use of microcirculation promoting and neuroprotective agents also encountered bottlenecks in clinical application. Therefore, the importance of reconstruction and repair of nerve function during the recovery period is gradually being recognized. Promoting neuro-restoration and regeneration attracts more and more attention of researchers and is gradually considered to be the key therapeutic strategy, including vascular regeneration/neovascularization, endogenous neural stem cell proliferation, differentiation and migration, axonal sprouting, and synaptic reconstruction.

The possibility of neuro-restoration is based on endogenous neurogenesis (Tang et al., 2023). Endogenous neurogenesis is the process by which endogenous neural stem/progenitor cells (NSCs/NPCs) are activated and differentiated into functional neurons, which usually occurs in the subventricular zone (SVZ) and the subgranular zone (SGZ) of the dentate gyrus (DG) of the hippocampus (Niklison-Chirou et al., 2020). It has been shown that NSCs present in specific regions of the mammalian brain are capable of continuous division and differentiation in the adult brain (Gage et al., 1998). Although this single process is not sufficient to fully repair the damaged brain, it still has an important meaning. Loss of synaptic activity is the earliest consequence of cerebral ischemia (Hofmeijer and van Putten, 2012), and damage to the pre-synaptic and post-synaptic membranes can be observed, in addition to signs of dissolution of synaptic structures after cerebral ischemia (Hofmeijer and van Putten, 2012). Synaptic plasticity is the ability of synaptic connections and signaling between neurons to change in response to changes in the body and external environment. This property of synapses makes it possible to re-establish functional circuits at nascent synapses after cerebral ischemia. Here, we focus on neurogenesis and synaptogenesis after cerebral ischemia.

Mitochondria play a vital role in the pathogenesis of ischemic stroke by regulating cellular energy metabolism, oxidative stress and subcellular apoptosis signaling pathways, therefore have an important impact on the occurrence and development of ischemic injury (Yang et al., 2018). Moreover, mitochondria have also been found in recent years to be closely associated with the process of brain tissue repair after ischemia (Brunetti et al., 2021). Mitochondria is a double-membrane organelle prevalent in eukaryotic cells and is the main source of energy for the organism. Routine neural activities such as transmission of neurotransmitter and the preservation of excitatory neuronal action potentials rely primarily on ATP’s production by mitochondria (Cobley et al., 2018). In addition, neurons maintain their own calcium homeostasis through mitochondria (Cardanho-Ramos and Morais, 2021). And calcium, as a universal intracellular second messenger, is highly involved in neuronal biochemical mechanisms, such as in the transmission of depolarization and synaptic activity, and enhances ATP production (Brini et al., 2014). It has also been found that synaptic mitochondria are more susceptible to calcium (Brown et al., 2006). The ultimate goal of neuro-restoration is to promote the recovery of neural function, and synaptic regeneration and synaptic reconstruction will ultimately affect the quality of post-stroke rehabilitation, in which mitochondria, as a cellular “calcium reservoir”, play a crucial role. Therefore, affecting mitochondrial function and improving mitochondrial morphology are potentially effective means to promote neuro-restoration.

Sonic hedgehog (Shh) pathway is one of the most studied hedgehog (HH) pathway. It plays an important role in juvenile cell development, proliferation, and in determining cell fate processes, while is relatively conserved in adulthood (Palma et al., 2005). The pathway is mainly composed of the Shh ligand, its receptor patched (Ptch1) and pathway activator smoothened (Smo), receptor transcription factors (Gli protein family) and the downstream target genes (Yao et al., 2016). When Shh is not present, Ptch binds to smo to inhibit Smo’s activity, thus inhibiting the expression of target genes; when Shh binds to Ptch, the inhibitory effect on Smo is lifted, and Smo is released, then transmitting the signal and activating the downstream Gli, which enters the nucleus and initiates the expression of a variety of target genes (Chen and Jiang, 2013). Current studies have shown that activation of the Shh signaling pathway promotes neurogenesis (Chen et al., 2018), especially in the hippocampus area. It has also been reported that mitochondrial proteins can be inhibited by activation of the Shh signaling pathway thereby reducing oxidative stress (Kaushal et al., 2018). All these evidences suggested that mitochondria may be the pivot in Shh signaling pathway and neurogenesis.

Catalpol (CAT), structurally belongs to the class of cyclic enol ether terpene glycosides, is one of the main active ingredients extracted from the Chinese herbal *Rehmannia glutinosa (Gaetn.) Libosch. ex Fisch. et Mey.* (Dihuang), which has the traditional effects of Nourishing *Yin* and Tonifying *Kidney*, Nourishing *Blood* and Tonifying *Blood*, and is extremely common used in clinical treatment of stroke (Jia et al., 2023a). Modern pharmacological studies have shown that CAT has certain neuroprotective effects in the acute phase of stroke through anti-inflammatory, antioxidant, and anti-apoptotic effects (Wang HJ. et al., 2022; Zhang et al., 2023). However, whether CAT can regulate mitochondrial function through the shh pathway to promote neuro-restoration has not been revealed. Therefore, in the present study, we investigated the neuro-restoration and regenerative effects of CAT and possible mechanisms by observing its effects on neurogenesis in the recovery period as well as morphological and functional changes in neurons.

## Materials and methods

### Materials

Catalpol (HPLC ≥ 98%) (2415-24-9) was bought from Shanghai Yuanye Bio-Technology Co., Ltd. (Shanghai, China). Ginaton (EGb761, EGb) were bought from Dr. Willmar Schwabe GmBH and Co. KG (Karlsruhe, Germany). Fluorescence microspheres (106–125 μm and 180–212 μmin diameter, UVPMS-BY2) were purchased from Cospheric (Goleta, United States). Goat serum (B900780) was purchased from Proteintech Group, Inc. (Wuhan, China). NeuroTraceTM530/615 red fluorescent Nissl stain (N21482), Goat anti-Rabbit IgG (H + L) Highly Cross-Adsorbed Secondary Antibody, Alexa Fluor™ Plus 488 (A32731) and Goat anti-Rat IgG (H + L) Cross-Adsorbed Secondary Antibody, Alexa Fluor™ 594 (A-11007) were purchased from Thermo Fisher Scientific Inc. (Waltham, United State). 4′,6-diamidino-2-phenylindole (DAPI) solution (C00650), TritonX100 (No.1109F0524) and 5-Bro-mo-2′-deoxyuridine (BrdU) (B8010-1) were purchased from Beijing Solarbio Science and Technology Co.,Ltd. (Beijing, China). Anti-BrdU Rat mAb (ab6326) and Anti-DCX Rabbit mAb (ab207175) were bought from abcam (Cambridge, United Kingdom). 4% Paraformaldehyde Fix Solution (G1101), Anti-Synaptophysin Rabbit pAb (GB11553), Anti -PSD95 Rabbit pAb (GB11277), Glutaraldehyde Fixed Solution (G1102) were bought from Wuhan Servicebio Technology CO.,Ltd. (Wuhan, China). Shh Rabbit pAb (bs1544R) was bought from Beijing Biosynthesis Biotechnology CO.,Ltd. (Beijing, China). HRP-Goat Anti-Rabbit IgG (H + L) (HX 2031) was bought from Huaxingbio (Beijing, China). Human/Mouse/Rat BDNF (brain derived neurotrophic factor) ELISA Kit (PB070), Total Antioxidant Capability Assay Kit with a Rapid ABTS method (S0121) were bought from Beyotime Biotechnology (Shanghai, China), Tissue Calcium Colorimetric Assay Kit (GMS50097.2) was purchased from GENMED SCIENTIFICS INC. U.S.A (MA, United States).

### Experimental animal

Male 8-week-old Sprague-Dawley (SD) rats (weight 210 ± 10 g) were provided by SPF (BEIJING) BIOTECHNOLOGY CO., LTD. (Beijing, China). All rats were housed in a specific pathogen-free facility at Xiyuan Hospital Animal Center with controlled temperature (22°C ± 2°C) and humidity (55% ± 5%), with a 12-h light/dark cycle, and free access to water and food. The experimental protocol was approved by the Experimental Ethics Committee of Xiyuan Hospital (2024XLC001-1). The rats were adapted for 3 days prior to the experiment. To assess the effect of CAT on neurogenesis and synaptogenesis in rats, the multiple cerebral infarction model was established by intracerebral injecting microsphere through unilateral internal carotid.

### Establishment of multiple cerebral infarction (MCI) model

The MCI model was prepared with slight modifications based on previous research in our laboratory (Gao et al., 2022). In short, the rats were fastened in the supine position after anesthesia by intraperitoneal injection of 80 mg/kg pentobarbital sodium. The right common carotid artery (CCA), internal carotid artery (ICA) and external carotid artery (ECA) were isolated and exposed, then the right pterygopalatine arteries were twisted off by electric coagulation pen and the right ECA were ligated with strings. Next, the right CCA for the rats were temporarily occluded with vascular clamp. Fluorescence microspheres, suspended in 200 ul of 5% dextran solution, were injected into the right ICA through a tip-blunted 22 Gauge syringe needle inserted into the ECA, then the vascular clamp occluding CCA was simultaneously removed, allowing the microspheres to move to the arteries of the brain randomly and lead to micro-embolisms, and then the wound at the neck was closed by sutures. The sham rats received equal volume of vehicle without microspheres.

### Grouping and drug treatment

Rats were grouped according to neurological deficit scores to minimize differences between groups the second day: sham group, model group, Ginkgo biloba P.E (EGb) (Ginaton:18 mg/kg) group, model + CAT 30 mg/kg group (CAT 30), model + CAT 60 mg/kg group (CAT 60), and model + CAT 120 mg/kg group (CAT 120).

From the first day to the 14th day after MCI, rats were given the corresponding doses of drugs by gastric administration every day (1 mL/100 g), and equal volumes of saline were given to both the sham group and model group.

### BrdU labeling

BrdU labeling is a method to detect the proliferative state of cells. BrdU is a thymine analog that is copied into cells when they proliferate. BrdU can be inserted into replicated DNA double strands in place of thymine nucleosides, and this substitution can be stabilized and carried over to the offspring cells. The cells are then fixed and denatured to detect the amount of BrdU in the DNA, which can be used to determine the cell’s proliferative capacity. After the cells have been fixed and denatured, the amount of BrdU in the DNA can be detected immunologically to evaluate the proliferative capacity of the cells.

**FIGURE 1:**
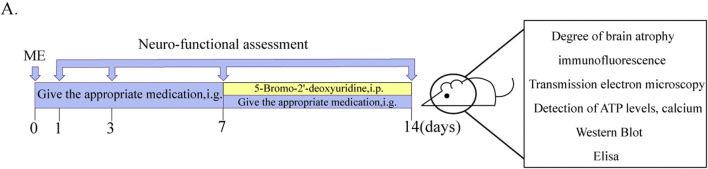
Schematic diagram of experimental delivery and testing protocols. **(A)** Diagram of experimental protocols.

From day 8 to day 14, all rats were injected with Brdu solution (50 mg/kg) i. p. The neurologic deficit scores were performed once each on postoperative days 1, 3, 7, and 14. After 2 h of last dose, the rats were euthanized, and the brain and plasma were collected. The experimental program is shown in Figure 1A.

### Neuro-functional assessment

The neurological deficit score was rated from 0 to 4 (0, no neurological deficit symptoms; 1, unable to completely stretch left forepaw; 2, circling to the left; 3, falling to the left or rolling on the ground; 4, no spontaneous activity with consciousness disorder) (Garcia et al., 1995; Wu et al., 2019). Rats with the score between one and three were included in the following experiments.

### Measurement of brain atrophy

On the 14th day after surgery, the rats were anesthetized and their brains were removed, irradiated under ultraviolet light and the pictures were recorded, and the area of the healthy hemibrain and the area of the ischemic hemibrain were measured using ImageJ. The degree of brain atrophy = area of the ischemic hemibrain/area of the healthy hemibrain × 100%.

### Perfusion and section

To minimize errors from other factors, some rats will undergo perfusion and section on the day of sample collection. After blood collection, the abdominal aorta was clamped with surgical forceps, and the chest cavity is opened. Next, 150–200 mL of Phosphate buffer saline (PBS) was drawn, and a needle was inserted at the apex of the heart. After cutting the right atrial appendage, perfusion was initiated. Once 1× PBS perfusion completes, 100–150 mL of 4% paraformaldehyde was injected until the rat’s skin turns pale and the limbs become stiff. Finally, the brain was quickly extracted and immersed in 15 mL of 4% paraformaldehyde at 4°C overnight. The next day, the fixative was replaced with a 25% sucrose solution and stored at 4°C until the brain sunk. The brains were then embedded and frozen in optimal cutting temperature compound (OCT compound) and a series of brain coronal sections (40 μm) were cut at −14°C using a cryomicrotome (Leica, Germany, CM 1950). Those brains will be used for fluorescent Nissl staining and immunofluorescence.

### Fluorescent nissl staining

In order to investigate the protective function of CAT on neuronal cells, the morphology and distribution of Nissl labeled neurons in rat brain was detected by fluorescent Nissl staining (Munoz-Ballester et al., 2022).

For Fluorescent Nissl Staining, brain sections were rinsed with 0.1 M Phosphate buffer (PB) for 5 min, and then incubated in a 0.1 M PB solution containing NeuroTrace™ 530/615 red fluorescent Nissl stain (1:2000), DAPI (1:100), 1% Triton X-100 in 0.1 M PB [rabbit anti-Nissl monoclonal antibody (1:1500)] overnight at 4°C and protected from light. On the second day, the sections were rinsed three times with 0.1 M PB for 5 min each in dark, mounted on a glass slide, sealed with 50% (v/v) glycerol, and then observed under a fluorescence microscope.

Images of hippocampal DG, Cornu Ammonis (CA) 2/CA3 and cortical regions were captured (Olympus, Olympus BX53, Japan) with a magnification of ×200. Afterwards, the number of Nissl-labeled neurons in the slices was counted according to the number of images taken (three animals per group, three coronal sections per animal were taken).

### Immunofluorescence

In order to investigate the effect of CAT on the differentiation potential towards neuron from NPCs, the expression of BrdU^+^/DCX^+^ cells in the cerebral hippocampus were detected by double immunofluorescence staining. Brain slices were obtained and processed as in *Perfusion and Section*. For BrdU/DCX double immunofluorescence staining, brain sections were first rinsed with 0.1 M PB for 5 min, then incubated in 2 M hydrochloride (HCl) at 37°C for 45 min to denature the DNA, and then incubated with 0.1 M sodium borate buffer (pH 8.5) for 10 min at room temperature, followed by rinsing with 0.1 M PB three times for 5 min each. Brain sections were then incubated in 0.1 M PB blocking solution containing 3% goat serum and 1% TritonX-100 for 30 min at room temperature, and then incubated overnight at 4°C in a solution containing 1% goat serum, 1% Triton X-100 and primary antibody [rabbit anti-DCX monoclonal antibody (1:100), rat anti-BrdU polyclonal antibody (1:500). The Second day, Sections were rinsed three times with 0.1 M PB for 5 min each time, and then incubated with the corresponding secondary antibody [DyLight 488-labeled goat anti-rabbit IgG (H&L) (1:1000), Dylight 594-labeled goat anti-rat IgG (H + L) (1:1000)] and DAPI(1:100) for 1.5 h at room temperature in the dark. After rinsing with 0.1 M PB, the brain sections were mounted on a glass slide, sealed with 50% (v/v) glycerol, and then observed.

Observational and statistical methods were the same as Fluorescent Nissl Staining, except that Brdu/DCX was observed only in the DG region.

### Enzyme-linked immunosorbent assay

The BDNF concentrations in the serum of rats after centrifugation (3,000 rpm/min, 15 min, 4°C) were measured using commercial ELISA kits (Human/Mouse/Rat Brain Derived Neurotrophic Factor Enzyme-Linked ImmunoSorbent Assay Kit, Beyotime Biotechnology) based on the manufacturer’s instructions.

### Tissue calcium colorimetric assay

The calcium ion concentration in the brain tissue of rats after cleaving and centrifugation (3,000 rpm/min, 15 min, 4°C), and the supernatant was taken, then using a commercial Calcium Colorimetric Assay Kit (Beyotime Biotechnology) and according to the manufacturer’s instructions.

### Total antioxidant capability assay

The Total Antioxidant Capability in the serum of rats after centrifugation (3,000 rpm/min, 15 min, 4°C) was detected by using a commercial Total Antioxidant Capability Assay Kit (Beyotime Biotechnology) and according to the manufacturer’s instructions.

### Transmission electron microscopy (TEM)

The brains of rats were removed on the 14th day after surgery, and the brains were rapidly removed on ice, and the hippocampus and cortex were separated from the lesion side hemispheres, and brain tissues of about 1 mm^2^ size were taken from hippocampal CA1 area, which were preserved in electron microscope fixative at 4°C. They were then rinsed three times with 0.1 M PBS for 15 min each time. Afterwards, they were fixed with 1% osmium acid, dehydrated with an ethanol gradient, and embedded. Ultrathin sections (80 nm) were made and then stained (2% dicumyl acetate and lead citrate). Finally, the morphology of mitochondria and synapses in the hippocampal region was observed under transmission electron microscope, and the images were captured by electron microscope camera.

### Western blot

Eighteen rats (3 rats per group) were decapitated and executed, and their brains were rapidly removed and frozen in liquid nitrogen. The ipsilateral hippocampus was isolated and homogenized with Radio Immunoprecipitation Assay (RIPA) lysate containing phosphatase inhibitor, protein phosphatase inhibitor and Phenylmethylsulfonyl fluoride (PMSF) (1:100) in a homogenizer at 4°C to thoroughly cleave the proteins, and then centrifuged to collect the supernatant. The protein content was detected by Bradford method, and the protein concentration was adjusted to the same level after quantification. Then, a certain concentration of sample solution was prepared by mixing with 5×sample buffer and separated by SDS-PAGE, and then the samples were transferred to polyvinylidene difluoride (PVDF) membranes (Millipore, United States). The membrane was blocked with 5% skimmed milk for 2 h at room temperature followed by incubated in primary antibodies [Shh (1:1000), SYP (1:1200), PSD-95 (1:2000), and β-actin (1:1000)] respectively at 4°C overnight. The membrane was then washed three times with Phosphate Buffered Saline with Tween 20 (PBST) for 10 min and incubated with the corresponding secondary antibodies for 1 h at room temperature. The immunoreactive bands were detected by BeyoECL Moon reagent (China) and the average gray value of each band was calculated by ImageJ software. β-actin was used as an internal control for Shh, SYP and PSD-95.

### Quantitative real-time polymerase chain reaction PCR (q-PCR)

Weigh 20–30 mg of ipsilateral hippocampal tissue and add TRIzol reagent (Thermo Fisher, 15596026) at a ratio of 1 mg tissue to 10 μL TRIzol. Homogenize the tissue, then add 1/5 chloroform and vortex the mixture, and incubate at room temperature for 15 min. Subsequently, centrifuge the mixture at 12,000 rpm for 15 min at 4°C. Carefully transfer 200 μL of the upper aqueous phase to a new tube and add an equal volume of isopropanol. Mix the contents and incubate at −20°C for 30 min. Centrifuge again at 12,000 rpm for 10 min at 4°C. Discard the supernatant and add 1 mL of 75% ethanol to each tube, followed by centrifugation at 12,000 rpm for 5 min at 4°C. Carefully remove the supernatant and invert the tubes to air dry at room temperature for 10 min. Finally, dissolve the RNA pellet in 20 μL of RNase-free water and incubate at room temperature for 10 min. Gently pipette the solution up and down several times to ensure complete dissolution. Take 3.5 μL of this solution and mix it with 3.5 μL of milli-Q water for RNA concentration and purity determination using a SMA-1000 nano-drop spectrophotometer. After removing the genomic DNA (gDNA) from the RNA samples, the RNA was then reverse transcribed into cDNA using the TOYOBO FSQ-201 ReverTra Ace qPCR RT Master Mix (TOYOBO, China). The cDNA was amplified using the SYBR^®^ Select Master Mix (Applied Biosystems, United States) and specific primers in the ABI StepOnePlus real-time PCR detection system (Applied Biosystems, United States). The primers (General Biosystems, China) are listed in Table 1. Relative mRNA expressions were calculated using the 2^-△△Ct^ method. All target genes were standardized with β-actin.

**TABLE 1:** Primer Sequences of rat for q-PCR.

Primer	Sequence (5′-3′)	Primer length (bp)
Gli1	Forward ACAGCGGCGTGGAGATGG	107
	REVERSE GCGGCGAAGGGTGGAGAG	107
β-actin	Forward TGC​TAT​GTT​GCC​CTA​GAC​TTC​G	240
	REVERSE GTT​GGC​ATA​GAG​GTC​TTT​ACG​G	240

### Statistical analysis

All data were expressed as mean ± standard deviation and analyzed with appropriate statistical methods using SPSS 21.0 software (IBM, Chicago, United States). If the data did not conform to a normal distribution, a nonparametric *U*-test was used to assess the differences between the two groups. Statistical comparisons between multiple groups were performed using one-way ANOVA or two-way repeated measures ANOVA followed by LSD test.

## Results

### CAT alleviates behavioral disorder and brain atrophy in MCI rats

**FIGURE 2:**
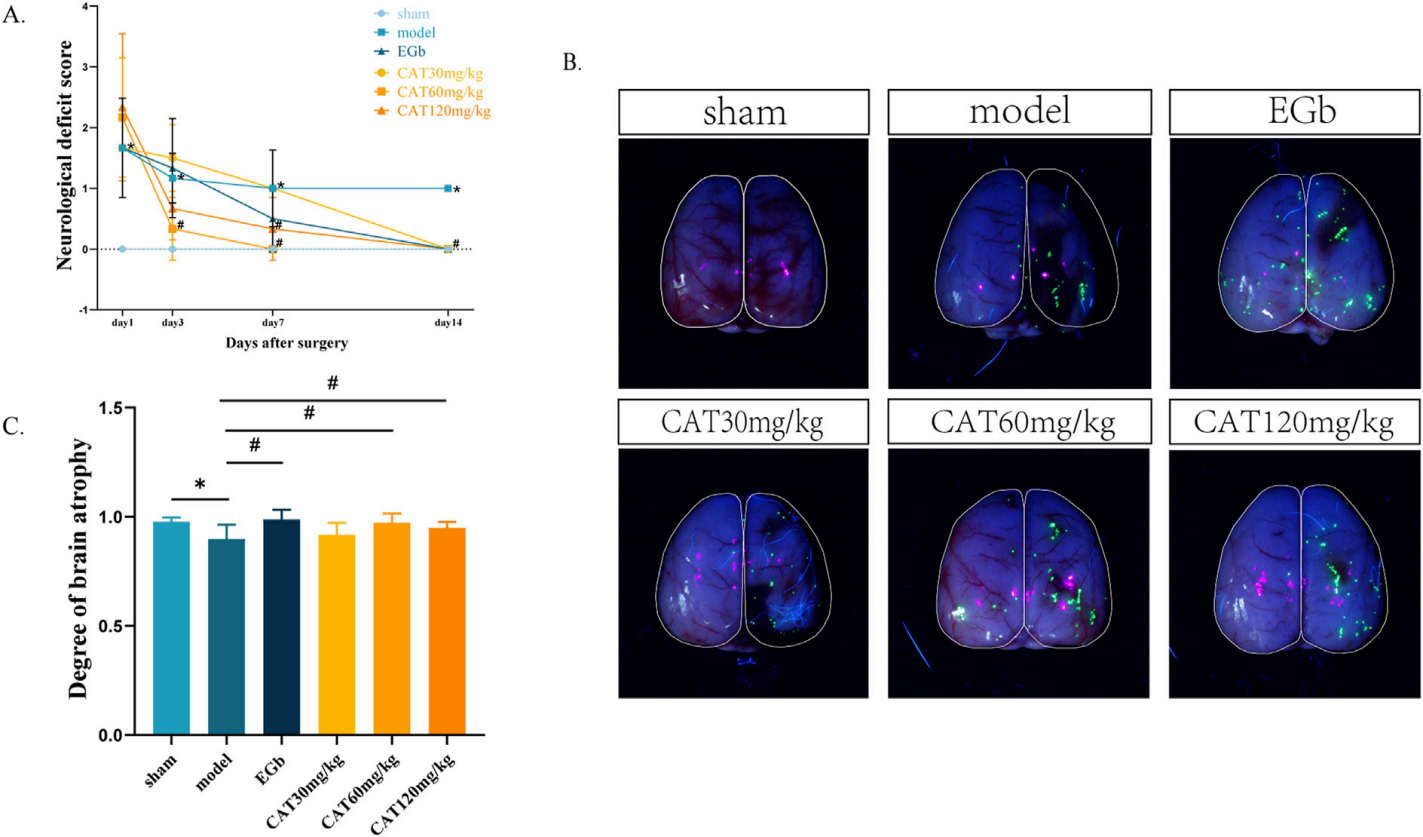
Effects of CAT on neurological deficit score and brain atrophy in MCI rats. **(A)** Neurological deficit score. **(B)** Representative pictures of brain atrophy. **(C)** Degree of brain atrophy. Those data were presented as mean ± SD (n = 8). *p < 0.05 compared with the control group. ^#^ *p* < 0.05 compared with the model group.

The neuro-restoration effect of CAT on rats with cerebral ischemia was assessed by behavioral and cerebral atrophy degree as shown in Figures 2A–C. Longa score is a common method of scoring neurological function in rats, and the higher the number, the more severe the neurological deficits, and the more likely that the rats would have the typical symptoms of stroke, such as hemiparetic numbness, difficulty in walking, and incoordination of movement. The neurologic deficit score in the model group was significantly higher than that in the sham group on day 1 post-stroke (P < 0.05), indicating successful modeling. On day 3, the score in the CAT 60 mg/kg group was significantly lower than that in the model group (P < 0.05). On day 7, it was also observed that the score of CAT 120 mg/kg group was significantly lower than that of the model group (P < 0.05). At day 14, both the EGb group and the administered group demonstrated a significant decrease in scores from the model group (P < 0.05).

During the recovery period after stroke, a severely damaged brain is likely to exhibit pathologically relevant atrophy, with greater atrophy indicating more severe brain damage. Here, we expressed the degree of brain atrophy as the percentage of damaged hemibrain area/normal hemibrain area, with smaller numbers indicating more severe brain atrophy. As shown in Figures 2B, C, after 14 days, the affected hemi-brain in the model group was significantly reduced compared to the sham group (P < 0.05), further indicating the success of modeling. The EGb group, the CAT60 mg/kg and CAT 120 mg/kg groups showed significant improvement compared to the model group (P < 0.05).

### CAT attenuates pathological damage in the hippocampus and cortex of MCI rats

**FIGURE 3:**
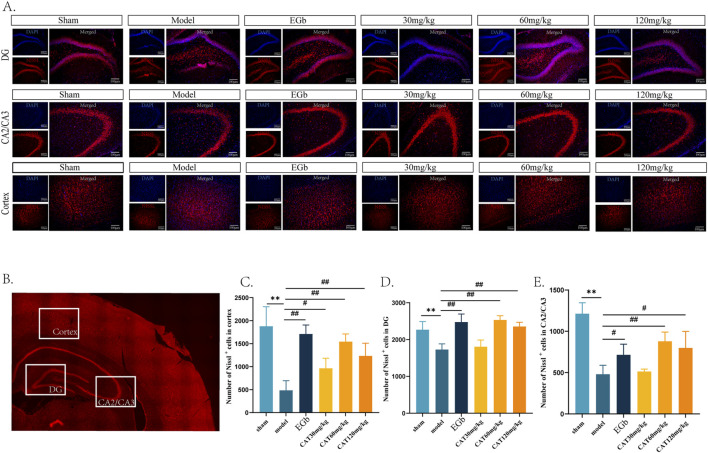
Pathologic effects of CAT on the hippocampus and cortex of MCI rats. **(A)** Representative pictures of six groups of Nissl labeled neurons in the DG, CA2/CA3 area and cortex (200x). **(B)** Schematic representation of the specific locations in a cross-section of the brain. **(C)** Number of Nissl labeled neurons in DG. **(D)** Number of Nissl labeled neurons in CA2/CA3. **(E)** Number of Nissl labeled neurons in cortex. These data were presented as mean ± SD (n = 3). *p < 0.05, **p < 0.01 compared with the control group. #*p* < 0.05, ##*p* < 0.01compared with the model group.

Next, we observed the hippocampal DG, CA2/CA3, and cortex areas of MCI rats stained by Nissl. Nissl body is one of the characteristic structures of neurons, and under normal physiological conditions, nerve cells exhibit large and numerous Nissl body. However, in neuronal damage, the number of Nissl bodies may decrease or even disappear. We observed that the number of Nissl labeled neurons in the cortex, CA2/CA3, and DG region in the model group (Figures 3A–E), which was highly significantly decreased (P < 0.01). And after administration, EGb group highly significantly upregulated the number of Nissl^+^ neurons in the cortex and DG regions (P < 0.01) and significantly promoted in the CA2/CA3 region (P < 0.05). And the CAT 120 mg/kg group had similar modulatory effects on the three regions as the EGb group. The CAT 60 mg/kg group also highly significantly upregulated the number of Nissl^+^ neurons in these three regions (P < 0.01). In addition, the CAT 30 mg/kg group also exhibited a significant boosting effect on cells in the cortex (P < 0.05).

### CAT promotes neurogenesis and upregulates BDNF expression

**FIGURE 4:**
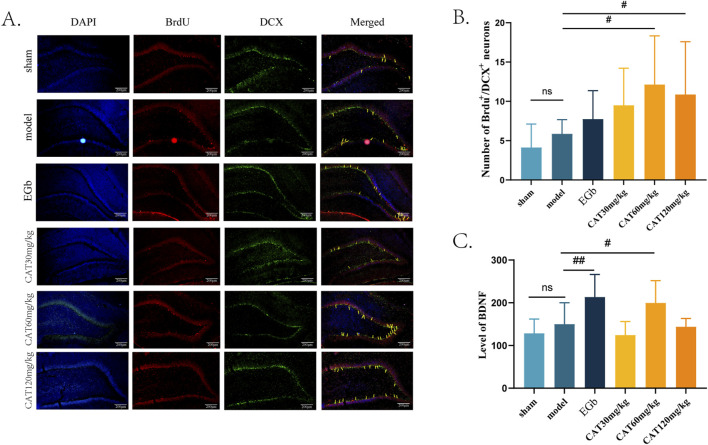
*Effect of CAT on brain neurogenesis and BDNF.* **(A)** Representative pictures of BrdU^+^/DCX^+^ cells in the DG region (200x). **(B)** Number of BrdU^+^/DCX^+^ cells (n = 8). **(C)** Level of BDNF (n = 7). These data were presented as mean ± SD. *p < 0.05 compared with the control group. ^#^ *p* < 0.05, ^##^ *p* < 0.01compared with the model group.

After observing the neuro-restoration effect of CAT, we further confirmed the pro-neurogenic effect of CAT by immunofluorescence staining and ELISA. In this experiment, we mainly analysed the captured images of the number of BrdU^+^/DCX^+^ cells in DG (Figure 4). DCX is one of the specific indicators of immature neurons, and the BrdU^+^/DCX^+^ cells mean the newborn immature neurons, which are manifested in the fluorescence layer as yellow or orange cells. When the number of yellow cells is higher, indicating that more NSCs proliferate and differentiate into neurons after cerebral ischemia and thus can be used as a measure of neurogenesis. Figure 4A shows that after cerebral ischemia, there is a trend that the number of BrdU^+^/DCX^+^ cells increase in DG of the rats, but there is no significant difference (P > 0.05). In contrast, the CAT 60 mg/kg and 120 mg/kg groups significantly upregulated the expression of BrdU+/DCX + cells (P < 0.05), and the CAT 60 mg/kg group showed a higher trend.

Meanwhile, we examined the expression of BDNF in the serum of MCI rats (Figure 4C). BDNF is an endogenous factor involved in neurogenesis in the central nervous system (CNS), especially in the hippocampal region, and an increase in its level after cerebral ischemia may indicate that the nerve cells are in a more favorable environment for recovery. Our results showed that the level of BDNF was upregulated in MCI rats after cerebral ischemia, but no significant difference was observed when compared to the sham group (P > 0.05). EGb group highly significantly increased the level of BDNF (P < 0.01). CAT 60 mg/kg group also significantly increased the BDNF level (P < 0.05).

### CAT promotes synaptogenesis

**FIGURE 5:**
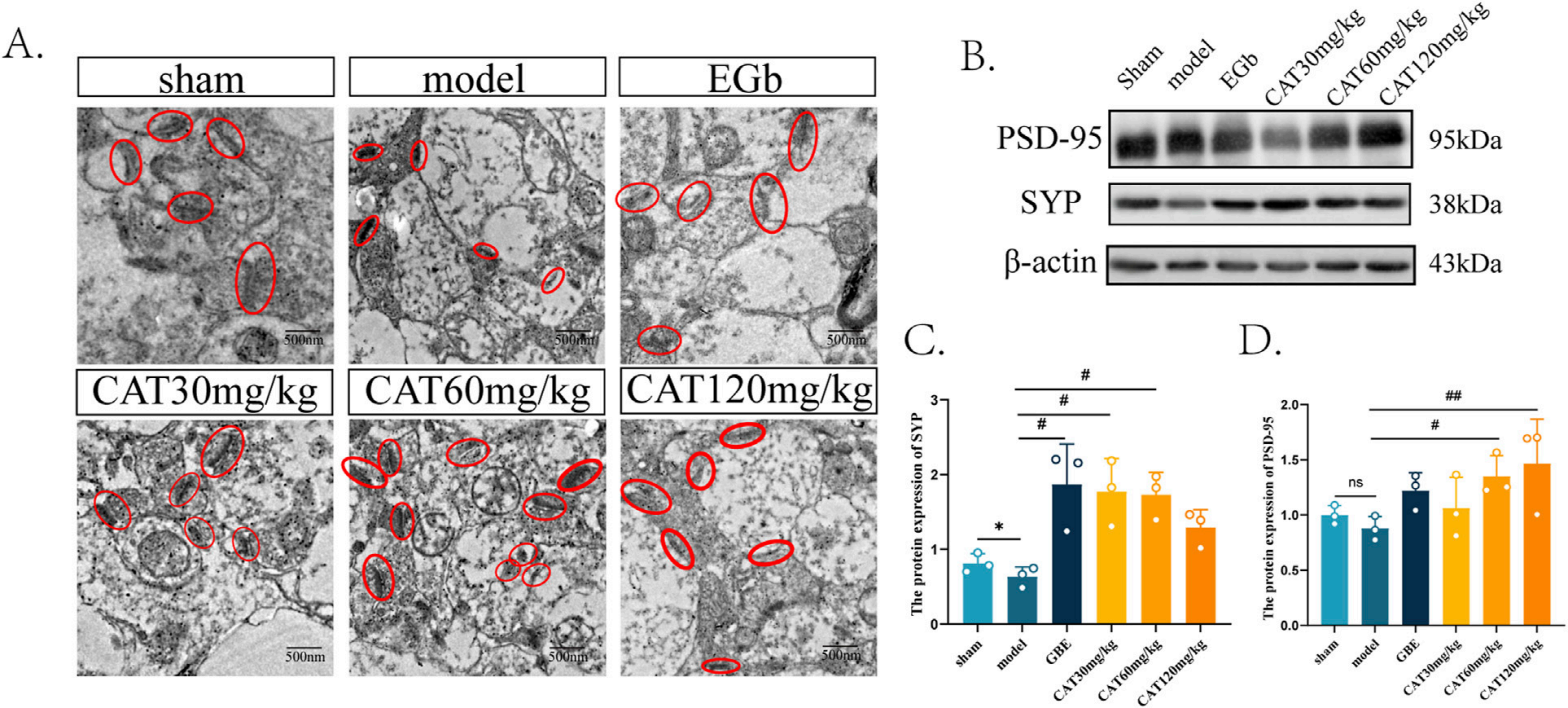
Effect of CAT on synaptogenesis. **(A)** Representative images of synapses from each group under TEM (250,00x). **(B)** The protein expressions of PSD-95, SYP were detected by Western blot. **(C)** The protein expression of SYP. **(D)** The protein expression of PSD-95. These data were presented as mean ± SD (n = 3). *p < 0.05 compared with the control group. #*p* < 0.05, ##*p* < 0.01compared with the model group.

We were also interested in whether CAT could further promote synaptogenesis in MCI rats, so we next observed the morphology and number of synapses under transmission electron microscope first. As shown in Figure 5A, the synaptic structure in Sham group was clear, normal and moderate in number, while in model group, the synaptic gap was extremely narrow and fuzzy, indicating that its structure had been severely damaged. And after administration, the EGb group clearly could be observed that the synaptic structure became clear, the synaptic gap became larger, and synaptic vesicles could be observed. All of the synaptic structures were somewhat improved after CAT administration, and more synapses were observed in the CAT 60 mg/kg group, indicating that synaptogenesis and connectivity may be promoted more at this dose.

Next, we tried to observe more evidence of CAT promoting synaptogenesis at the level of molecular biology. As shown in Figures 5B–D, the protein expression of PSD-95 as well as SYP decreased after modeling, and there was a significant difference in the protein expression of SYP (P < 0.05), suggesting that there were different degrees of structural damage and reduction after cerebral ischemia. After administration, the EGb group significantly up-regulated the protein expression of SYP (P < 0.05), and there was a tendency to rise on the aspect of PSD-95 protein expression. The CAT 30 mg/kg group and CAT 60 mg/kg group significantly upregulated the protein level of SYP (P < 0.05), and also the CAT 60 mg/kg group significantly promoted the expression of PSD-95 protein (P < 0.05). The CAT 120 mg/kg group had a highly significant upregulation of PSD-95 protein (P < 0.01), while it did not significantly promote the expression of SYP.

### CAT improves mitochondrial structure and function after brain injury

**FIGURE 6:**
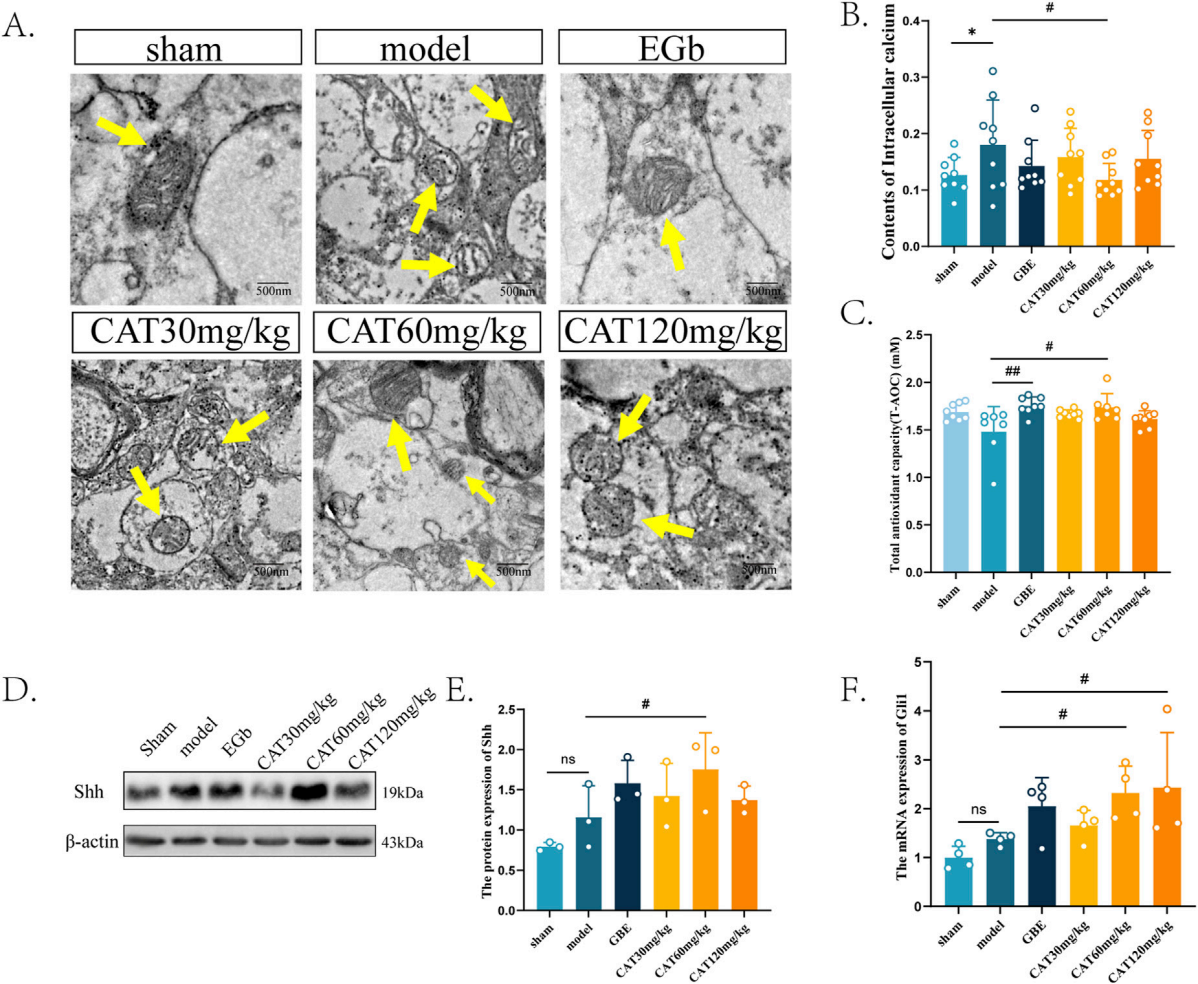
Effects of CAT on mitochondria, and an involvement of Shh signaling pathway. **(A)** Representative images of each group of mitochondria under TEM (250,00x). **(B)** Contents of Intracellular calcium (n = 9). **(C)** Total antioxidant capability of each group (n = 7). **(D)** The protein expression of Shh was detected by Western blot in the cortex of MCI rats. **(E)** The protein expression of SHH (n = 3). **(F)** The mRNA expression of Gli1 (n = 4). These data were presented as mean ± SD. *p < 0.05 compared with the control group. #*p* < 0.05 compared with the model group.

Next, we observed the effects of CAT on the structure and function of mitochondria in the brain tissue of MCI rats. As shown in Figure 6A, the mitochondria in the Sham group were observed to have a normal rod shape under TEM, and the number of cristae was higher, with smaller cristae gaps and no vacuolization, and the mitochondrial matrix was richer. In the Model group, the mitochondria were observed to have a rounded globular morphology and the number of cristae was reduced, with severe vacuolation. The mitochondrial cristae in the EGb group had an increase in the number of cristae with smaller cristae spacing, and there was no vacuolization, indicating that the morphological structure of mitochondria was improved. CAT 30 mg/kg group mildly increased the number of mitochondrial cristae, but there was still vacuolization. Similarly, CAT 120 mg/kg group increased the number of cristae and reduced vacuolization. On top of that, the CAT 60 mg/kg group also enriched the inner mitochondrial matrix, suggesting that CAT administration also had an ameliorating effect on mitochondrial structure.

**FIGURE 7:**
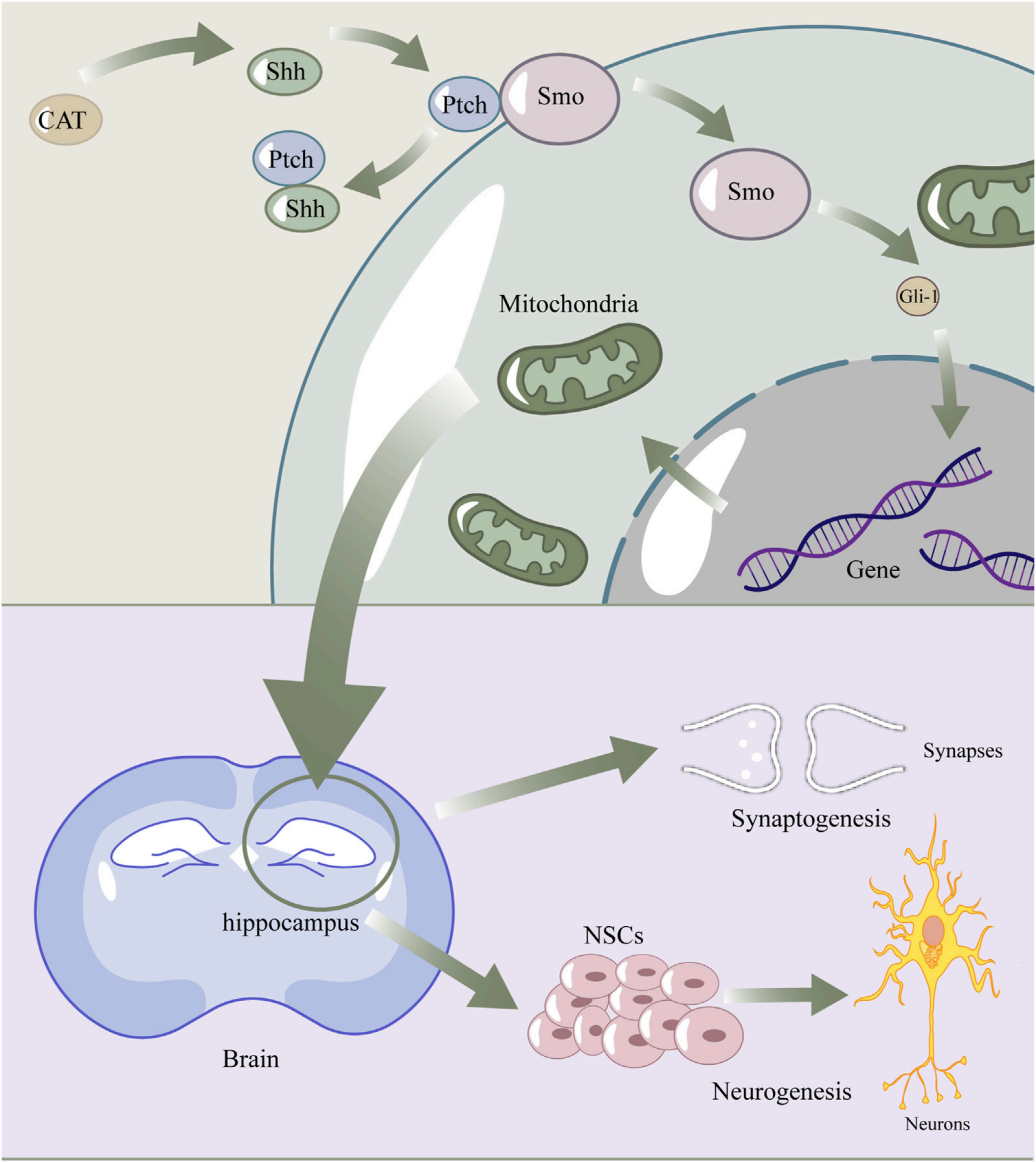
The diagram of catalpol enhance Shh signaling pathway to promote neurogenesis and synaptogenesis by regulating mitochondria. Catalpol upregulated shh, then shh connected with ptch, which normally combined with smo. Smo was released to activate Gli-1. Gli-1 got into the nuclear and affect expression of proteins, which lead to the recovery of mitochondrial structure and fuction. Finally, these promoted Synaptogenesis and Neurogenesis.

Next, we observed the restoration of mitochondrial function by detecting the intracortical calcium content and T-AOC in the cerebral cortex of MCI rats (Figures 6B, C). Mitochondrial calcium homeostasis is an important indicator of mitochondrial function. Under normal conditions, mitochondria can maintain the dynamic balance of calcium ions in intracellular content, and after ischemia occurs, the decrease in mitochondrial membrane potential leads to calcium ion efflux (Garbincius and Elrod, 2022), resulting in intracellular calcium overload, which may ultimately lead to cell death. Therefore, abnormally elevated intracellular calcium levels imply, to a certain extent, impaired mitochondrial function. Our experiments found that the intracortical calcium content in the rat cortex was significantly elevated after modeling (P < 0.05), whereas the CAT 60 mg/kg group significantly reduced the intracellular calcium content (P < 0.05), and the other CAT groups also showed a tendency to reduce the calcium content. This implies that CAT can improve mitochondrial function.

Reactive oxygen species (ROS), primarily generated by mitochondrial respiration, act as a “double-edged sword.” At physiological levels, ROS are widely involved in cellular signal transduction and vital processes. However, after a stroke, excessive ROS production can trigger mitochondrial oxidative stress, leading to aging and related diseases (Tönnies and Trushina, 2017). An increase in T-AOC may indicate the restoration of mitochondrial function. Similarly, by assessing the T-AOC in each group after the stroke, We observed a significant improvement in the catalpol-treated group (60 mg/kg) in T-AOC (Figure 6F).

### CAT activates the shh signaling pathway

Last, we examined the protein and mRNA expression of shh signaling pathway-related members at the protein and gene levels, respectively. The results, as shown in Figures 6C–E, showed that the protein expression of Shh as well as the mRNA expression of Gli1 were slightly elevated, but did not express significant differences (P > 0.05). Similarly in these two indicators, the EGb group also showed a rising trend, but also did not perform a significant difference (P > 0.05), which suggests that the EGb may not exert its therapeutic effect on neurological disorders through the Shh pathway. After administration, the CAT 60 mg/kg group significantly upregulated the protein expression of Shh as well as the mRNA expression of Gli1 (P < 0.05). The mRNA expression of Gli1 was also significantly elevated in the CAT 120 mg/kg group (P < 0.05). This suggests that CAT may improve the mitochondrial structure and architecture through shh signaling pathway, thus achieving the purpose of neuro-restoration.

## Discussion

The pathological mechanisms of cerebral ischemia are complex. After the ischemic event, oxygen and glucose supply is absent, which impairs mitochondrial function involved in energy metabolism. This, in turn, leads to an alteration of the mitochondrial membrane potential and, at the same time, to the activation of voltage-dependent calcium channels and an imbalance in calcium homeostasis, leading to intracellular calcium overload. In addition to this, impaired mitochondrial function leads to the release of reactive oxygen species, also the microglia activation (Fang et al., 2023). All these contribute to the processes of excitotoxicity, inflammatory response, oxidative stress and apoptosis, which finally lead to cell death (Maurya et al., 2022). The discovery of NSCs has provided new ideas and strategies for the treatment of brain injury. After a stroke, the brain initiates some self-help mechanisms in which endogenous NSCs proliferate, differentiate and migrate to the infarcted area, followed by synaptic neogenesis, axonal sprouting, and re-establishing contact with those undamaged neurons. The use of drugs to intervene in this process is a very promising therapeutic direction in the field of neuro-restoration.

In previous studies of catalpol, we have found that it contributes to save neurons both in the acute phase after stroke and in the recovery phase, and involves different mechanisms. It is reported that intranasal administration of catalpol can protect rats from acute cerebral ischemia through mechanisms such as antioxidant stress and anti-apoptosis. (Wang J. et al., 2022). During the recovery phase, which is our main focus, there is substantial evidence suggesting that catalpol promotes angiogenesis and encourages the differentiation of neural stem cells, potentially through multiple pathways, including the upregulation of VEGF production (Wang HJ. et al., 2022; Dong et al., 2016; Sun et al., 2023). Moreover, *in vitro* studies have demonstrated catalpol’s anti-inflammatory effects on neuroinflammation, possibly by downregulating pro-inflammatory cytokines such as IL-6, TNF-α, and IL-1β, and effectively inhibiting nitric oxide production (She et al., 2024). It is well-established that neurogenesis in the adult hippocampus is influenced by various intrinsic factors, including neuroinflammation, aging, and oxidative stress (Poulose et al., 2017). Current research indicates that mitochondrial components and metabolic byproducts play a role in modulating inflammatory processes (Marchi et al., 2023). On the other hand, oxidative stress has been widely recognized as a contributing factor in the aging process and the progression of several neurodegenerative diseases, including Alzheimer’s disease. Increased production of reactive oxygen species (ROS) is linked to mitochondrial dysfunction and a decline in antioxidant defenses with age, which directly impacts neuronal synaptic activity (Tönnies and Trushina, 2017). Although there is limited evidence regarding the influence of the Shh signaling pathway on processes like inflammation and oxidative stress, its strong association with mitochondrial function suggests that the Shh pathway may regulate key processes involved in neural repair through mitochondria as intermediaries.

In this study, we used a non-classical method of cerebral infarction modeling, in order to ensure the survival rate of rats and the success rate of infarction. The effectiveness of this modeling method in exploring physiopathological mechanisms in ischemic brain injury or neural regeneration has been demonstrated by many research teams around the world over decades (Shen et al., 2022; Miyake et al., 1993; Kisoh et al., 2017), where the distribution of fluorescent microspheres on the surface of the cerebral cortex as well as in coronal sections was observed within 6 h after injection, and these distribution patterns were similar. Furthermore, we took the immunofluorescence staining method and demonstrated that there was significant damage to the brain cells of rats after modeling. This method was also used in other studies (Zhang et al., 2019; Gao et al., 2024; Wang et al., 2020), confirming that microsphere-induced ischemia is suitable for studies in the area of drug-promoted neuro-restoration mechanisms.

In this study, we investigated the neurorestorative effects of CAT in rats after MCI, and attempted to approach its molecular mechanism of action associated with mitochondrial morphology and function.

Fourteen days after MCI, we found that CAT improved the neurological function, and reduced the degree of brain atrophy, suggesting that CAT has a neurological recovery effect in the of cerebral ischemia. In the observations of behavior, we found that the CAT group was able to reduce the rats’ neurological deficit scores more rapidly, as observed on the third day of assessment, whereas the EGb demonstrated a more subdued downward trend, consistent with its performance in the clinic (Li et al., 2017). In our study, both EGb and CAT groups presented some pro-restorative effects to the brain. Brain atrophy refers to a reduction in brain volume as well as enlargement of the ventricles due to age or other diseases, and is usually observed in the late stages of stroke. The exact mechanism of brain atrophy remains unclear, but the degree of atrophy reflects the severity of brain damage (Veldsman, 2017).

Nissl body is usually found in the cytoplasm of neurons, and the Nissl labeled cells can be clearly observed about the size and structure of the neurons, as well as their distribution (Uylings et al., 1999). Our study found that neurons distributed in both cortex and hippocampus after MCI showed a wrinkled shape, scattered arrangement of cortical neurons, and a significant decrease in number. After administration of CAT, the neurons were found to be full in shape, the cortical neurons were neatly arranged, and their number was significantly increased. This suggests that CAT can ameliorate the damage caused by ischemia to nerve cells.

We also found that CAT significantly increased the number of BrdU^+^/DCX^+^ cells, suggesting that CAT promotes the differentiation of nascent NSCs into early neuronal cells during stroke recovery. Neurogenesis in post-adulthood occurs mainly in the SVZ of the lateral ventricle and the SGZ. In the present study, we focused on NSCs proliferation and differentiation in the dentate gyrus of the hippocampus through immunofluorescent staining. Brdu, an analog of the endogenous DNA base thymine, which can easily insert into DNA during cytosolic s phase of cell division can be readily inserted into DNA and used to determine whether cells are proliferating (Cavanagh et al., 2011). DCX, on the other hand, is a microtubule-associated protein that is commonly used to label immature neurons (Tobin et al., 2019). Here, we distinguished which were newborn immature neurons by cells successfully labeled by both Brdu and DCX, and judged neurogenesis in the hippocampal dentate gyrus based on the level of double-positive cell counts. Immunofluorescence results showed that CAT significantly promoted the proliferation of NSCs in the hippocampal region and their differentiation into neurons.

BDNF is a class of proteins that play an important role in the survival, differentiation and functional maintenance of neurons in the central and peripheral nervous systems, and is closely related to neural neogenesis and synaptogenesis (Barde, 1989). It has been shown that BDNF levels are significantly low in the serum of rats with cerebral ischemia (Chen et al., 2012). In the hippocampal region, the BDNF content of rats in the ischemic group was significantly lower than that of rats in the sham-operated group (Karantali et al., 2021). Our experiments revealed that CAT can increase the expression of BDNF, which enables the damaged nerve cells to live in a relatively favorable microenvironment, and implied to us that the mechanism of CAT-promoted neuro-restoration may also be related to BDNF.

Next, we looked further at newly produced synapses. Mature neurons are highly differentiated, as evidenced by the differentiation of axons and dendrites, and the regulation of synaptic plasticity is essential for the construction of neural circuits (Wu et al., 2022). Healthy synapses should exhibit clear synaptic spacing and synaptic vesicles. Under the observation of TEM, in addition to the number of synapses, we also observed the structure of synapses. The blurring of synaptic spacing in the rats of the model group proved that the structure of synapses was dissolved after ischemia. CAT can increase the number of synapses and improve the synaptic structure to a certain extent. SYP is a presynaptic marker and PSD-95 is a postsynaptic marker, both of which are closely related to synapse formation and neurotransmission (Cousin, 2021; Ugalde-Triviño and Díaz-Guerra, 2021). SYP is specifically distributed in the vesicular membrane, and may be involved in synaptic vesicle formation and cytosolic emesis, but the specific mechanism is not explored (Hoffmann et al., 2021). PSD-95 is the most abundantly present in the post-synaptic membrane, and also one of the most important proteins, involved in the regulation of development. PSD-95 is one of the most abundant and important proteins on the postsynaptic membrane, which is involved in regulating the number of synapses during development and promoting synapse formation (Kim and Sheng, 2004). In chemical synapses, the anterior membrane of secretory synaptic vesicles relies on Ca^2+^ influx to activate voltage-gated Ca^2+^ channels, which in turn allows rapid emptying of neurotransmitters into the synaptic gap. This process requires action potential triggering. In turn, the maintenance of electrochemical gradients, synaptic vesicle secretion and recirculation are all energy-intensive processes (Devine and Kittler, 2018). It is well known that mitochondria are cellular calcium reservoirs and are involved in the regulation of neuronal calcium homeostasis (Olson et al., 2012). For this purpose, mitochondria are dispersed by neurons to sites closer to the synapse (Li et al., 2004). Here, we found that CAT significantly reduced calcium levels and upregulated the protein expression of SYP and PSD-95 at both *in vitro* and *in vivo* levels, suggesting that CAT promotes the functional restoration of mitochondria, which in turn regulates calcium homeostasis and enhances synaptic plasticity.

In the post-ischemic brain, we also observed changes in the morphological structure of mitochondria and found that CAT reduced mitochondrial vacuolization and increased the number of cristae. Normal mitochondria should exhibit an oval shape, rod-like in three-dimensional structure, abundant matrix within the mitochondria, and a large and compact number of cristae (Protasoni and Zeviani, 2021). Damaged mitochondria, on the other hand, show a shape close to spherical or even fragmented. In our experiments, CAT helped to reduce calcium levels, which represents an improvement in mitochondrial function by CAT. Those improvements on mitochondria may had a connection with Shh signaling pathway, as the pathway was also observed activation meanwhile.

It is known that the Shh pathway is essential for neuronal development, maturation, and the formation of neural circuits, as are mitochondria, but little is known about the link between the two. What is available now suggests that taurine enhances ATP production, reduces reactive oxygen species content, and stabilizes mitochondrial membrane potential, all of which are blocked by shh pathway inhibitors (Jia et al., 2023b). In addition, Shh pathway activation was found to increase mitochondrial membrane potential and respiratory activity, and may be affected by inhibition of Drp1 (Kaushal et al., 2018). In our study, CAT clearly activated the shh pathway, as seen by the observation of protein expression of shh and mRNA expression of Gli1. This suggests the Shh pathway affects mitochondria directly or indirectly, but the specific mechanism through which the Shh pathway affects mitochondria is not yet clear.

Our results also showed that CAT 60 mg/mL had the optimal effect within the three dose groups of CAT based on the overall situation analysis (Table 2), which is consistent with previous studies (Ming et al., 2011; Xiaoshuang et al., 2017; Shengwei et al., 2013). In addition, our study also set up a more concentrated concentration group and found that the effect was not as good as that of the medium dose group, which suggests that the quantitative effect curve of CAT is not always positively proportional to the trend, and that we are probably close to the concentration of the optimal effect, but more experiments are needed to verify exactly how it is.

**TABLE 2:** Summary table of the effect of each CAT group on different indicators.

Possible mechanisms		CAT (mg/kg)		
		30	60	120
Improving Neurological Deficit	Neurological Deficit		↓↓	↓
Reducing brain atrophy			↓	↓
Improving Neuron Morphology	Hippocampus DG		↑↑	↑↑
	Hippocampus CA2/CA3		↑↑	↑
	Cortex	↑	↑↑	↑↑
Improving Neurogenesis	BrdU+/DCX + cells		↑	↑
	Improving BDNF levels		↑	
Improving Synaptogenesis	SYP·Protein	↑	↑	
	PSD-95·Protein		↑	↑↑
Improving mitochondrial Deficit of structure and Dysfuction	T-AOC		↑	
	Calcium		↑	
Activation of the shh signaling pathway	Shh·Protein		↑	
	Gli1·mRNA		↑	↑

“↑” or “↓” indicates a significant increase or decrease, “↑↑” or “↓↓” indicates a highly significant increase or decrease.

Our study still has some limitations. We used the nonclassical method of modeling, and unlike the classical method MCAO, the location of microsphere occlusion was not subject to human manipulation after injection of microspheres from the ICA. As a result, the infarcted area was more diffuse and the size of the infarcted area was uncertain. While this implies a closer resemblance to multiple cerebral infarcts, at the same time, the behavioral manifestations will be less pronounced, as the blockage of micro-vessels does not necessarily severely damage brain regions related to learning, memory, or limb control. The method of inducing cerebrovascular occlusion using microspheres was first proposed in 1990s (Takagi et al., 1997). In previous studies conducted by our research group, various behavioral assessments of MCI rats after multiple cerebral infarctions (2 weeks) were performed, including the Neuro-Functional Assessment, Forepaw Outreaching Test, and Rope-Climbing Test, all of which showed statistically significant differences compared to the sham group (Gao et al., 2022). Furthermore, direct evidence was obtained demonstrating that after the injection of fluorescent microspheres, they could be visualized on the brain surface and in brain slices using fluorescent stereomicroscopy, revealing significant histopathological changes in the brain slices (Shen et al., 2022). Therefore, we believe that this model remains suitable for investigating neuro-restoration in rats with multiple cerebral infarctions.

Cerebral ischemia causes irreversible damage to nerve cells, which means that the functions responsible for the corresponding brain areas are also permanently impaired, leading to paralysis of limbs, or loss of learning and memory abilities, and possible complications such as dementia and depression. Neuro-restoration is particularly necessary. Our study demonstrates that CAT is able to promote endogenous neurogenesis and modulate synaptic plasticity on the basis of a certain neuroprotective effect. It is possible that this effect is achieved by modulating the structure and function of mitochondria through the shh pathway. Nevertheless, how CAT activates the shh pathway and how its downstream factors affect various aspects of mitochondria need to be explored in the future.

## Conclusion

In conclusion, our study demonstrated that oral administration of CAT enhanced the recovery of neurologic function, improved the degree of brain atrophy, and enhanced endogenous neurogenesis and synaptogenesis, ameliorating pathological damage in the hippocampus and cortex of the rat brain after cerebral infarction. CAT upregulates the expression of shh proteins and thus improves mitochondria, which may be a potential mechanism for the aforementioned effects of CAT (Figure 7). It is noteworthy that BDNF is closely linked to mitochondria, the Shh signaling pathway, and neuro-restoration, and is likely to be an intermediate mediator of this link. Our study provides additional evidence for the pharmacodynamics of CAT, as well as some new insights into the connection between the shh pathway and mitochondria.

## Data Availability

The original contributions presented in the study are included in the article/supplementary material, further inquiries can be directed to the corresponding authors.

## References

[B1] Barde Y. A. (1989). Trophic factors and neuronal survival. Neuron 2 (6), 1525–1534. 10.1016/0896-6273(89)90040-8 2697237

[B2] Brini M. Calì T. Ottolini D. Carafoli E. (2014). Neuronal calcium signaling: function and dysfunction. Cell Mol. Life Sci. CMLS 71 (15), 2787–2814. 10.1007/s00018-013-1550-7 24442513 PMC11113927

[B3] Brown M. R. Sullivan P. G. Geddes J. W. (2006). Synaptic mitochondria are more susceptible to Ca2+overload than nonsynaptic mitochondria. J. Biol. Chem. 281 (17), 11658–11668. 10.1074/jbc.M510303200 16517608

[B4] Brunetti D. Dykstra W. Le S. Zink A. Prigione A. (2021). Mitochondria in neurogenesis: implications for mitochondrial diseases. Stem Cells Dayt Ohio 39 (10), 1289–1297. 10.1002/stem.3425 34089537

[B5] Cardanho-Ramos C. Morais V. A. (2021). Mitochondrial biogenesis in neurons: how and where. Int. J. Mol. Sci. 22 (23), 13059. 10.3390/ijms222313059 34884861 PMC8657637

[B6] Cavanagh B. L. Walker T. Norazit A. Meedeniya A. C. B. (2011). Thymidine analogues for tracking DNA synthesis. Mol. Basel Switz. 16 (9), 7980–7993. 10.3390/molecules16097980 PMC6264245 21921870

[B7] Chen A. Lin Z. Lan L. Xie G. Huang J. Lin J. (2012). Electroacupuncture at the Quchi and Zusanli acupoints exerts neuroprotective role in cerebral ischemia-reperfusion injured rats via activation of the PI3K/Akt pathway. Int. J. Mol. Med. 30 (4), 791–796. 10.3892/ijmm.2012.1074 22842715

[B8] Chen S. D. Yang J. L. Hwang W. C. Yang D. I. (2018). Emerging roles of sonic hedgehog in adult neurological diseases: neurogenesis and beyond. Int. J. Mol. Sci. 19 (8), 2423. 10.3390/ijms19082423 30115884 PMC6121355

[B9] Chen Y. Jiang J. (2013). Decoding the phosphorylation code in Hedgehog signal transduction. Cell Res. 23 (2), 186–200. 10.1038/cr.2013.10 23337587 PMC3567827

[B10] Cobley J. N. Fiorello M. L. Bailey D. M. (2018). 13 reasons why the brain is susceptible to oxidative stress. Redox Biol. 15, 490–503. 10.1016/j.redox.2018.01.008 29413961 PMC5881419

[B11] Cousin M. A. (2021). Synaptophysin-dependent synaptobrevin-2 trafficking at the presynapse-Mechanism and function. J. Neurochem. 159 (1), 78–89. 10.1111/jnc.15499 34468992

[B12] Devine M. J. Kittler J. T. (2018). Mitochondria at the neuronal presynapse in health and disease. Nat. Rev. Neurosci. 19 (2), 63–80. 10.1038/nrn.2017.170 29348666

[B13] Dong W. Xian Y. Yuan W. Huifeng Z. Tao W. Zhiqiang L. (2016). Catalpol stimulates VEGF production via the JAK2/STAT3 pathway to improve angiogenesis in rats’ stroke model. J. Ethnopharmacol. 191, 169–179. 10.1016/j.jep.2016.06.030 27301615

[B14] Fang J. Wang Z. Miao C. Y. (2023). Angiogenesis after ischemic stroke. Acta Pharmacol. Sin. 44 (7), 1305–1321. 10.1038/s41401-023-01061-2 36829053 PMC10310733

[B15] Gage F. H. Kempermann G. Palmer T. D. Peterson D. A. Ray J. (1998). Multipotent progenitor cells in the adult dentate gyrus. J. Neurobiol. 36 (2), 249–266. 10.1002/(sici)1097-4695(199808)36:2<249::aid-neu11>3.0.co;2-9 9712308

[B16] Gao J. Liu J. Yao M. Zhang W. Yang B. Wang G. (2022). Panax notoginseng saponins stimulates neurogenesis and neurological restoration after microsphere-induced cerebral embolism in rats partially via mTOR signaling. Front. Pharmacol. 13, 889404. 10.3389/fphar.2022.889404 35770087 PMC9236302

[B17] Gao J. Yao M. Zhang Y. Jiang Y. Liu J. (2024). Panax notoginseng saponins stimulates the differentiation and neurite development of C17.2 neural stem cells against OGD/R injuries via mTOR signaling. Biomed. Pharmacother. Biomedecine Pharmacother. 172, 116260. 10.1016/j.biopha.2024.116260 38382327

[B18] Garbincius J. F. Elrod J. W. (2022). Mitochondrial calcium exchange in physiology and disease. Physiol. Rev. 102 (2), 893–992. 10.1152/physrev.00041.2020 34698550 PMC8816638

[B19] Garcia J. H. Wagner S. Liu K. F. Hu X. J. (1995). Neurological deficit and extent of neuronal necrosis attributable to middle cerebral artery occlusion in rats. Statistical validation. Stroke 26 (4), 627–634. 10.1161/01.str.26.4.627 7709410

[B20] Hoffmann C. Sansevrino R. Morabito G. Logan C. Vabulas R. M. Ulusoy A. (2021). Synapsin condensates recruit alpha-synuclein. J. Mol. Biol. 433 (12), 166961. 10.1016/j.jmb.2021.166961 33774037

[B21] Hofmeijer J. van Putten MJAM (2012). Ischemic cerebral damage: an appraisal of synaptic failure. Stroke 43 (2), 607–615. 10.1161/STROKEAHA.111.632943 22207505

[B22] Jia J. Chen J. Wang G. Li M. Zheng Q. Li D. (2023a). Progress of research into the pharmacological effect and clinical application of the traditional Chinese medicine Rehmanniae Radix. Biomed. Pharmacother. Biomedecine Pharmacother. 168, 115809. 10.1016/j.biopha.2023.115809 37907043

[B23] Jia J. Tian X. He J. Ma G. He W. (2023b). Taurine promotes axonal sprouting via Shh-mediated mitochondrial improvement in stroke. Acta Cirúrgica Bras. 38, e382323. 10.1590/acb382323 PMC10292808 37377249

[B24] Karantali E. Kazis D. Papavasileiou V. Prevezianou A. Chatzikonstantinou S. Petridis F. (2021). Serum BDNF levels in acute stroke: a systematic review and meta-analysis. Med. Kaunas. Lith. 57 (3), 297. 10.3390/medicina57030297 PMC8004775 33809965

[B25] Kaushal J. B. Popli P. Sankhwar P. Shukla V. Dwivedi A. (2018). Sonic hedgehog protects endometrial hyperplasial cells against oxidative stress via suppressing mitochondrial fission protein dynamin-like GTPase (Drp1). Free Radic. Biol. Med. 129, 582–599. 10.1016/j.freeradbiomed.2018.10.427 30347228

[B26] Kim E. Sheng M. (2004). PDZ domain proteins of synapses. Nat. Rev. Neurosci. 5 (10), 771–781. 10.1038/nrn1517 15378037

[B27] Kisoh K. Hayashi H. Itoh T. Asada M. Arai M. Yuan B. (2017). Involvement of GSK-3β phosphorylation through PI3-K/akt in cerebral ischemia-induced neurogenesis in rats. Mol. Neurobiol. 54 (10), 7917–7927. 10.1007/s12035-016-0290-8 27866373 PMC5684253

[B28] Li S. Zhang X. Fang Q. Zhou J. Zhang M. Wang H. (2017). Ginkgo biloba extract improved cognitive and neurological functions of acute ischaemic stroke: a randomised controlled trial. Stroke Vasc. Neurol. 2 (4), 189–197. 10.1136/svn-2017-000104 29507779 PMC5829919

[B29] Li Z. Okamoto K. I. Hayashi Y. Sheng M. (2004). The importance of dendritic mitochondria in the morphogenesis and plasticity of spines and synapses. Cell 119 (6), 873–887. 10.1016/j.cell.2004.11.003 15607982

[B30] Marchi S. Guilbaud E. Tait S. W. G. Yamazaki T. Galluzzi L. (2023). Mitochondrial control of inflammation. Nat. Rev. Immunol. 23 (3), 159–173. 10.1038/s41577-022-00760-x 35879417 PMC9310369

[B31] Maurya S. K. Gupta S. Bakshi A. Kaur H. Jain A. Senapati S. (2022). Targeting mitochondria in the regulation of neurodegenerative diseases: a comprehensive review. J. Neurosci. Res. 100 (10), 1845–1861. 10.1002/jnr.25110 35856508

[B32] Ming L. Yang L. Yi Z. Jianning S. Jingshu T. Xuanzhen Y. (2011). Catalpol up-regulated NGF,BDNF and mRNA gene expression and improved behavior outcome of rats with ischemic stroke. China J. Tradit. Chin. Med. Pharm. 05 (26), 977–981.

[B33] Miyake K. Takeo S. Kaijihara H. (1993). Sustained decrease in brain regional blood flow after microsphere embolism in rats. Stroke 24 (3), 415–420. 10.1161/01.str.24.3.415 8446979

[B34] Munoz-Ballester C. Mahmutovic D. Rafiqzad Y. Korot A. Robel S. (2022). Mild traumatic brain injury-induced disruption of the blood-brain barrier triggers an atypical neuronal response. Front. Cell Neurosci. 16, 821885. 10.3389/fncel.2022.821885 35250487 PMC8894613

[B35] Niklison-Chirou M. V. Agostini M. Amelio I. Melino G. (2020). Regulation of adult neurogenesis in mammalian brain. Int. J. Mol. Sci. 21 (14), 4869. 10.3390/ijms21144869 32660154 PMC7402357

[B36] Olson M. L. Chalmers S. McCarron J. G. (2012). Mitochondrial organization and Ca2+ uptake. Biochem. Soc. Trans. 40 (1), 158–167. 10.1042/BST20110705 22260683

[B37] Palma V. Lim D. A. Dahmane N. Sánchez P. Brionne T. C. Herzberg C. D. (2005). Sonic hedgehog controls stem cell behavior in the postnatal and adult brain. Dev. Camb Engl. 132 (2), 335–344. 10.1242/dev.01567 PMC1431583 15604099

[B38] Poulose S. M. Miller M. G. Scott T. Shukitt-Hale B. (2017). Nutritional factors affecting adult neurogenesis and cognitive function. Adv. Nutr. Bethesda Md 8 (6), 804–811. 10.3945/an.117.016261 PMC5683005 29141966

[B39] Protasoni M. Zeviani M. (2021). Mitochondrial structure and bioenergetics in normal and disease conditions. Int. J. Mol. Sci. 22 (2), 586. 10.3390/ijms22020586 33435522 PMC7827222

[B40] Saini V. Guada L. Yavagal D. R. (2021). Global epidemiology of stroke and access to acute ischemic stroke interventions. Neurology 97 (20_Suppl. ment_2), S6–S16. 10.1212/WNL.0000000000012781 34785599

[B41] She Y. Shao C. Y. Liu Y. F. Huang Y. Yang J. Wan H. T. (2024). Catalpol reduced LPS induced BV2 immunoreactivity through NF-κB/NLRP3 pathways: an *in vitro* and *in silico* study. Front. Pharmacol. 15, 1415445. 10.3389/fphar.2024.1415445 38994205 PMC11237369

[B42] Shen Y. Yao M. J. Su Y. X. Xu D. S. Wang J. Wang G. R. (2022). Histochemistry of microinfarcts in the mouse brain after injection of fluorescent microspheres into the common carotid artery. Neural Regen. Res. 17 (4), 832–837. 10.4103/1673-5374.322470 34472483 PMC8530124

[B43] Shengwei Z. Shifen D. Ting W. Jianning S. (2013). Effects of catalpol on real-time gait during early recovery in rats with permanent cerebral ischemia. Mod. Tradit. Chin. Med. Mater Medica-World Sci. Technol. (08 vo 15), 1676–1681.

[B44] Sommer C. J. Schäbitz W. R. (2021). Principles and requirements for stroke recovery science. J. Cereb. Blood Flow. Metab. Off. J. Int. Soc. Cereb. Blood Flow. Metab. 41 (3), 471–485. 10.1177/0271678X20970048 PMC7907998 33175596

[B45] Sun S. Xu Y. Yu N. Zhang M. Wang J. Wan D. (2023). Catalpol alleviates ischemic stroke through promoting angiogenesis and facilitating proliferation and differentiation of neural stem cells via the VEGF-A/KDR pathway. Mol. Neurobiol. 60 (11), 6227–6247. 10.1007/s12035-023-03459-9 37439957

[B46] Takagi N. Miyake K. Taguchi T. Tamada H. Takagi K. Sugita N. (1997). Failure in learning task and loss of cortical cholingergic fibers in microsphere-embolized rats. Exp. Brain Res. 114 (2), 279–287. 10.1007/pl00005636 9166917

[B47] Tang H. Li Y. Tang W. Zhu J. Parker G. C. Zhang J. H. (2023). Endogenous neural stem cell-induced neurogenesis after ischemic stroke: processes for brain repair and perspectives. Transl. Stroke Res. 14 (3), 297–303. 10.1007/s12975-022-01078-5 36057034

[B48] Tobin M. K. Musaraca K. Disouky A. Shetti A. Bheri A. Honer W. G. (2019). Human hippocampal neurogenesis persists in aged adults and Alzheimer’s disease patients. Cell Stem Cell 24 (6), 974–982. 10.1016/j.stem.2019.05.003 31130513 PMC6608595

[B49] Tönnies E. Trushina E. (2017). Oxidative stress, synaptic dysfunction, and Alzheimer’s disease. J. Alzheimers Dis. Jad. 57 (4), 1105–1121. 10.3233/JAD-161088 28059794 PMC5409043

[B50] Ugalde-Triviño L. Díaz-Guerra M. (2021). PSD-95: an effective target for stroke therapy using neuroprotective peptides. Int. J. Mol. Sci. 22 (22), 12585. 10.3390/ijms222212585 34830481 PMC8618101

[B51] Uylings H. B. Zilles K. Rajkowska G. (1999). Optimal staining methods for delineation of cortical areas and neuron counts in human brains. NeuroImage. 9 (4), 439–445. 10.1006/nimg.1999.0417 10191172

[B52] Veldsman M. (2017). Brain atrophy estimated from structural magnetic resonance imaging as a marker of large-scale network-based neurodegeneration in aging and stroke. Geriatr. Basel Switz. 2 (4), 34. 10.3390/geriatrics2040034 PMC6371114 31011044

[B53] Wang H. J. Ran H. F. Yin Y. Xu X. G. Jiang B. X. Yu S. Q. (2022a). Catalpol improves impaired neurovascular unit in ischemic stroke rats via enhancing VEGF-PI3K/AKT and VEGF-MEK1/2/ERK1/2 signaling. Acta Pharmacol. Sin. 43 (7), 1670–1685. 10.1038/s41401-021-00803-4 34795412 PMC9253350

[B54] Wang J. Zhang Y. Zhang M. Sun S. Zhong Y. Han L. (2022b). Feasibility of catalpol intranasal administration and its protective effect on acute cerebral ischemia in rats via anti-oxidative and anti-apoptotic mechanisms. Drug Des. Devel Ther. 16, 279–296. 10.2147/DDDT.S343928 PMC8801896 35115763

[B55] Wang M. Yao M. Liu J. Takagi N. Yang B. Zhang M. (2020). Ligusticum chuanxiong exerts neuroprotection by promoting adult neurogenesis and inhibiting inflammation in the hippocampus of ME cerebral ischemia rats. J. Ethnopharmacol. 249, 112385. 10.1016/j.jep.2019.112385 31730888

[B56] Wu T. Jia Z. Dong S. Han B. Zhang R. Liang Y. (2019). Panax notoginseng saponins ameliorate leukocyte adherence and cerebrovascular endothelial barrier breakdown upon ischemia-reperfusion in mice. J. Vasc. Res. 56 (1), 1–10. 10.1159/000494935 30763928

[B57] Wu Y. K. Miehl C. Gjorgjieva J. (2022). Regulation of circuit organization and function through inhibitory synaptic plasticity. Trends Neurosci. 45 (12), 884–898. 10.1016/j.tins.2022.10.006 36404455

[B58] Xiaoshuang Z. Jianning S. Liming B. (2017). Influence of catalpol on the learning and memory abilities and the expression of Bax and Bcl-2 protein in hippocampus of vascular dementia rats. West China J. Pharm. Sci. (01 vo 32), 40–42. 10.13375/j.cnki.wcjps.2017.01.013

[B59] Yang J. L. Mukda S. Chen S. D. (2018). Diverse roles of mitochondria in ischemic stroke. Redox Biol. 16, 263–275. 10.1016/j.redox.2018.03.002 29549824 PMC5854930

[B60] Yao P. J. Petralia R. S. Mattson M. P. (2016). Sonic hedgehog signaling and hippocampal neuroplasticity. Trends Neurosci. 39 (12), 840–850. 10.1016/j.tins.2016.10.001 27865563 PMC5148655

[B61] Zhang Y. Liu J. Yao M. Song W. Zheng Y. Xu L. (2019). Sailuotong capsule prevents the cerebral ischaemia-induced neuroinflammation and impairment of recognition memory through inhibition of LCN2 expression. Oxid. Med. Cell Longev. 2019, 8416105. 10.1155/2019/8416105 31565154 PMC6745154

[B62] Zhang Z. Dai Y. Xiao Y. Liu Q. (2023). Protective effects of catalpol on cardio-cerebrovascular diseases: a comprehensive review. J. Pharm. Anal. 13 (10), 1089–1101. 10.1016/j.jpha.2023.06.010 38024856 PMC10657971

